# Design, synthesis, and biological activity evaluation of dihydromyricetin derivatives against SARS-CoV-2-Omicron virus

**DOI:** 10.1080/14756366.2024.2390909

**Published:** 2024-08-29

**Authors:** Cong Wu, Qi Jiang, Hui Zhong, Xudong Zhou, Leping Liu, Tong Pan, Chao Liu, Wei Wang, Wenbing Sheng

**Affiliations:** a School of Pharmacy, Hunan University of Chinese Medicine, Changsha, Hunan, People’s Republic of China; b TCM and Ethnomedicine Innovation and Development International Laboratory, Hunan University of Chinese Medicine, Changsha, Hunan, People’s Republic of China; c Zhangjiajie Meicha Technology Research Center Hunan Qiankun Biotechnology Co., Ltd, Zhangjiajie, People’s Republic of China

**Keywords:** Dihydromyricetin, flavone, synthesis, SARS-CoV-2 3CL^pro^, Omicron virus BA.5

## Abstract

An oxidising and substituting one-pot reaction strategy has been developed to synthesise dihydromyricetin derivatives with the aim of enhancing the inhibitory activity of dihydromyricetin against SARS-CoV-2. Different *ω*-methoxy-*ω*-oxeylkyl was introduced in C_7_-OH site and yielded eight analogs, all of them showed good inhibitory activity against SARS-CoV-2 3CL^pro^ with IC_50_ values ranging from 0.72 to 2.36 μM. In the Vero E6-cell, compound **3** has a good activity of anti-SARS-CoV-2 virus (Omicron virus BA.5) in the prevention model, with an EC_50_ of 15.84 μM, and so do compound **10** in the therapeutic model, with an EC_50_ of 11.52 μM. The results suggest that the introduction of long chain *ω*-oxeylkyl at C_7_-OH facilitate the inhibition of viral replication in the therapeutic model, which is consistent with the binding energies predicted from molecular docking conclusions. It implies that dihydromyricetin derivatives have the potential to become effective inhibitors of SARS-CoV-2 Omicron and other viruses.

## Graphical abstract

**Figure:**
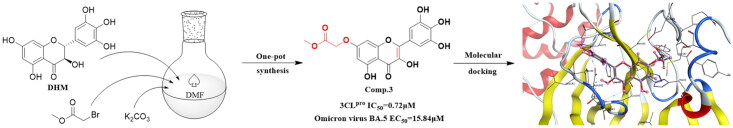

## Introduction

Severe acute respiratory syndrome coronavirus 2 (SARS-CoV-2)^1^^,^^2^, caused a global infectious coronavirus disease 2019 (COVID-19) and poses a significant threat to public health. Omicron virus is the major variants^3^ of SARS-Cov-2 mutated in the spike (S) gene^4^, which spread globally in 2021–2023^5^. Vaccine-activating antibodies have less activity against omega, and the risk of breakthrough infection remains high even after vaccination or infection with other variants^6^^,^^7^.

The Omicron variant with highly infectious and virulent was first identified in South Africa in late 2021^8^, and has been detected in more than 170 countries^5^. Which has more than eight sublineages including BA.1, BA.2, BA.2.12.1, BA.2.3, BA.2.9, BA.3, BA.4, and BA.5^9^^,^^10^ and has at least 36 new mutations in its spike (S) protein^9^^,^^11^, but RNA-dependent RNA polymerase (RdRp) and 3CL protease (3CL^pro^ or M^pro^) remain valid targets for the treatment of Omicron viruses^12^. Remdesivir, Molnupiravir, VV116, Obeldesivir, Ritonavir-Boosted Nirmatrelvir and Ensitrelvir are either being used urgently for Omicron control or in clinical trials^12^. The first FDA-approved drug for SARS-CoV-2, Remdesivir-resistance mutations still exist based on the Global Initiative on Sharing All Influenza Data (GISAID)^13^.

Although Omicron virus has multiple mutations in the S gene, the crucial 3CL^pro^ is not noticeably mutated^4^^,^^14^. SARS-CoV-2 is a single-stranded RNA virus, and its major protease is the 3CL protease^15^, it is responsible for cleaving 11 different sites of polyproteins to convert into mature functional proteins in SARS-CoV-2 replication^16^. Meanwhile, the protease homologous to 3CL^pro^ does not exist in humans, the inhibition of 3CL^pro^ hardly affects the normal physiological activities of the human body^17^, so 3CL^pro^ is still an ideal target for inhibiting SARS-CoV-2 Omicron virus. In this situation, the effective drugs used to prevent and treat the SARS-CoV-2 variant are still necessary.

Approximately 80% population in the world depends on medicinal plants or herbs for their medical needs^18^. Biological secondary products such as adenosine^19^, flavonoids^20^ and limonoids^21^ can exhibit antiviral effects in coronavirus infection models. For example, glycyrrhizin, withaferin A, curcumin, nigellidine and cordifolioside A were able to inhibit SARS-CoV-2 replication and reduce host inflammatory response^22^. Similar observations may be reported for phyto complexes^23^, such as *Paulownia tomentosa*^24^ extract that can be directly involved in the interaction between 3CL^pro^ and S protein, and the extract of *Citrus paradisi* Macfad^21^ that can exert antiviral activity in Vero E6 cells.

**Figure 1.**
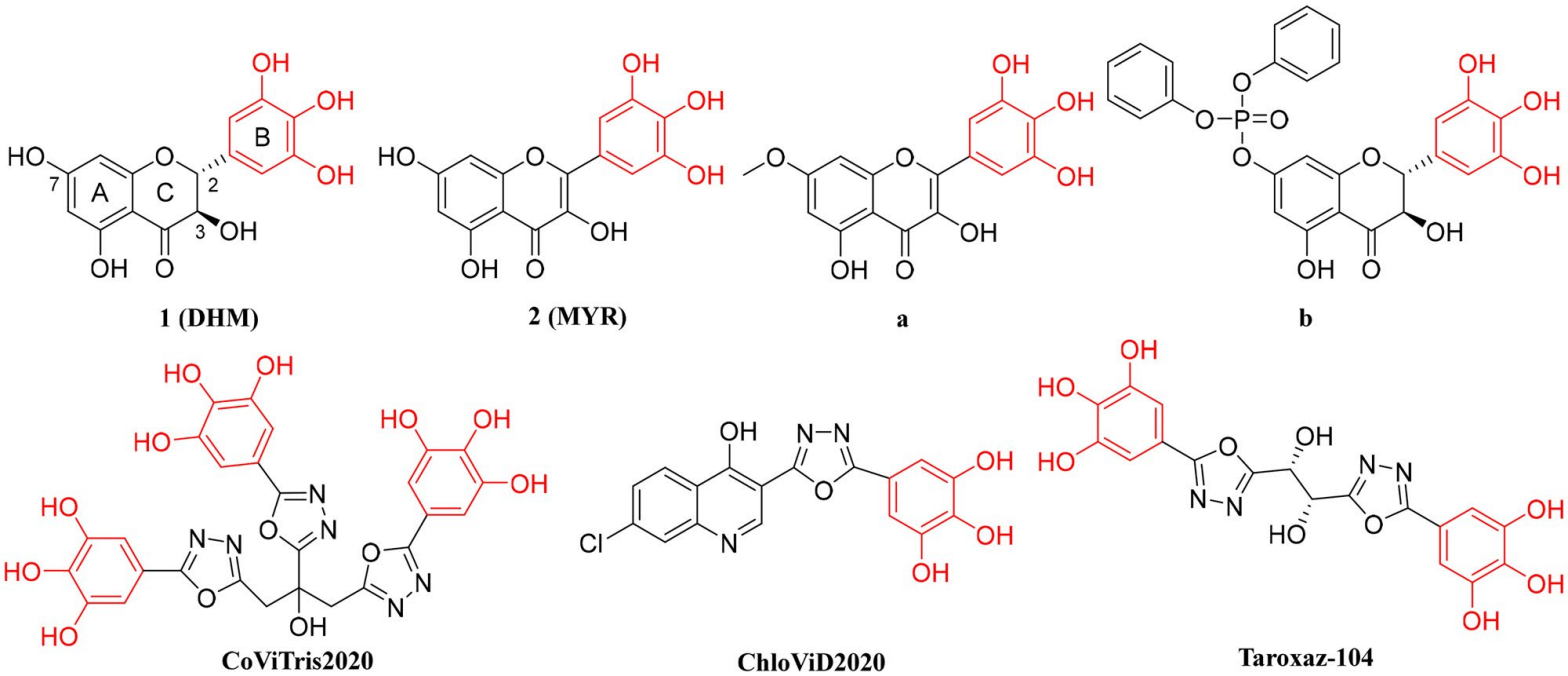
The chemical structure of dihydromyricetin, myricetin, compounds **a** and **b**, CoViTris2020, ChloViD2020, and Taroxaz-104.

Dihydromyricetin (Figure 1, DHM) is the highest flavonoid components of Meicha (*Ampelopsis grossedentata W. T. Wang*), with 30% content in its dry shoots^25^. It possesses a variety of biological activities, including antioxidant^26^^,^^27^, antiviral^28^, anticarcinogen^29^, antibacterial^30^, antifatigue^31^, antianxiety^32^, hepatoprotection^33^^,^^34^, cardioprotection^35^ and blood sugar regulation^36^^,^^37^. Recent studies have shown that DHM can inhibit SARS-CoV-2 by forming a covalent bond with 3CL^pro^ and has intracellular antiviral activity with EC_50_ value of 13.56 μM^38^. DHM has a high safety, and the results in acute oral toxicity tests^39^, long-term toxicity tests^40^ and genotoxicity tests^41^ are negative in rats and mice. Myricetin (Figure 1, MYR) is relatively more stable than DHM^42^, and it is proposed to enhance its antiviral activity on the basis of the oxidation of DHM to MYR.

DHM is widely used as a raw material for new drug development due to its special skeleton, extensive biological activity and availability^43–47^. And its derivatives **a** and **b** (Figure 1) showed strong inhibitory activity against SARS-CoV-2 3CL^pro^. Structure-activity relationship (SAR) suggests that the strong activity may be mainly due to the oxidation of the pyrogallol moiety of B ring and the oxidation of C-C bond between C_2_-C_3_. CoViTris2020 and ChloViD2020^48^^,^^49^ (Figure 1) have strong inhibition effect on original SARS-CoV-2 and high safety and the results showed that 3,4,5-trihydroxyphenyl moieties greatly increased the blocking affinities and potencies of SARS-CoV-2/human enzymes or proteins active sites and/or allosteric sites. Taroxaz-104^50^^,^^51^, the first reported anti-VOC-202012/01 agent, is a very promising potent nCoV-RdRp/SARS-CoV-2 inhibitor. It was again proved that 3,4,5-trihydroxyphenyl moieties is the pharmacophore of anti-SARS-CoV-2. However, derivatives **a** and **b** showed several-fold differences in the inhibitory activity of SARS-CoV-2 3CL^pro^ and SARS-CoV-2 transfected cells, which suggests that derivatives **a** and **b** are difficult to cross the cell membrane or may partially bind to other proteases after entry resulting in decreased activity^38^.

It has been proved that DHM and its derivatives have an inhibitory activity against the original strain of SARS-CoV-2. In order to enhance cell membrane penetration and anti-SARS-CoV-2 activity, SARS-CoV-2 Omicron virus was selected to evaluate the inhibitory effect of DHM and its derivatives. The synthetic design refers to the retention of the B-ring 3,4,5-trihydroxyphenyl structure and the introduction of lipophilic groups in the A-ring.

The σ bond between C_2_ - C_3_ provides a feasibility for the modification of DHM, and so do the C_7_-OH. Etherification of C_7_-OH and oxidation of the σ bond between C_2_ - C_3_ to a double bond were designed in a one-step reaction, and compounds **3**–**10** were obtained by this strategy. In order to investigate whether the chain length of the *ω*-oxeylkyl etherification product at the C_7_-OH would affect the inhibitory activity against SARS-CoV-2, cytotoxicity experiments of compounds **3–10** were designed to against SARS-CoV-2 3CL^pro^ by SARS-CoV-2 transfected cells and normal cells.

## Materials and methods

### General experimental procedures

Unless otherwise noted, all chemicals were obtained from commercial sources and used without further processing. DHM was obtained from Meicha which was authenticated as tender leaves of *Ampelopsis grossedentata W. T. Wang* by Prof. Wei Wang (voucher specimen: 2022042501). All other reagents were purchased from Aladdin Bio-Chem Technology Co., LTD. (Shanghai, the People’s Republic of China).^1^H NMR and ^13^C NMR spectra were measured with a Bruker 600 MHz NMR spectrometer. Thin-layer chromatography (TLC) was performed on silica gel HSGF-254 plates (0.20 ± 0.03 mm, Jiangyou Silica gel Development Co., LTD, Yantai, the People’s Republic of China). Vero E6 cells and Omicron virus BA.5 were obtained from National Virus Resource Centre, China.

### General procedure for the synthesis of 3–10

1.0 mmol DHM, 0.5 mmol potassium carbonate and 0.5 mmol Bromocarboxylate substituent were added to a 10 ml round flask charged with 2.0 ml DMF, stirred and refluxed at 60 °C for 12 h. After the reaction was complete, stopped the reaction and cooled it to room temperature,10% aqueous acetic acid was added and extracted with ethyl acetate (10 ml × 4). The organic layer was combined, and the solvent was removed in vacuum, the crude product was obtained. The crude product was purified by octadecyl silane chromatography (ODS) with the eluent (MeOH-H_2_O) to give the desired product.

### SARS-CoV-2 3CL^pro^ activity assay

The fluorescence resonance energy transfer (FRET) method was adapted to detect the enzyme activity. The inhibitory activity of DHM derivatives against SARS-CoV-2 3CL^pro^ was detected by using the SARS-CoV-2 3CL^pro^ Inhibitor Screening Kit (Enhanced) #P0315M (Beyotime, China)^52^. The whole experiment was carried out in a 96-well black microtiter plate with a total volume of 100 μL of liquid in each well, and three parallel wells were set up for each sample. Blank control wells were charged with 91 μL assay buffer, 5 μL DMSO and 4 μL substrate (MCA-AVLQSGFR-Lys(Dnp)-Lys-NH_2_); 100% enzyme activity control wells were charged with 90 μL assay buffer, 1 μL SARS-CoV-2 3CL^pro^, 5 μL DMSO, and 4 μL substrate; and the sample wells were homogeneously mixed with 90 μL assay buffer, 1 μL 3CL^pro^ and 5 μL DMSO-solubilised DHM derivatives (dilution concentrations of 0.25, 0.50, 1.00, 2.00, 4.00 μM) respectively; and the sample wells were incubated at 37 °C for 10 min. 4 μL of fluorescent substrate was quickly added to each well, and the fluorescence intensity was detected after shaking in a multifunctional enzyme labeller (Tecan Spark, Switzerland) for 15 s and incubating at 37 °C for 5 min, and the detecting maximal excitation wavelength of 325 nm and the maximal emission wavelength of 393 nm. Inhibition rate (%) = (RFU 100% enzyme activity control-RFU sample)/(RFU 100% enzyme activity control-RFU blank control) × 100%.

### Molecular docking

The docking module in Ledock.win32 software^53^ was used to perform docking simulations. The simulate docking results of a 3D crystal model of the protein structure of SARS-CoV-2 M^pro^/3CL^pro^ (PDB ID: 6LU7)^54^ with DHM derivatives was analysed. For successful docking, all water molecules were first removed from the protein in MOE 2019.1012 software^55^, and then hydrogen atoms were added to the protein using the LePro module in Ledock.win32 software with the docking pockets set to x = −18.6 min, −0.1 max; y = 6.5 min, 26.4 max; z = 58.2 min, 72.6 max, then the structural formula of the DHM derivatives was saved in 3D format (mol2.) and imported into the Ledock module for molecular docking, finally, the data corresponding to the simulated docking binding minimum values were imported into MOE to view the results of the compounds and proteins docking.

### Viral activity assay in Vero E6 cells

#### Preventive model

The cytotoxicity of the candidate compounds against Vero E6 cells was assessed using the CCK-8 assay. Cells were inoculated in 96-well plates (20 000 cells/well) and incubated at 37 °C in 5% CO_2_ for 24 h. Adding dihydromyricetin derivatives at different concentrations, and the cells would have further incubation for 24 h. The cell viability was determined by using 2–(2-Methoxy-4-nitrophenyl)-3–(4-nitrophenyl)-5–(2,4-disulfophenyl)-2H-tetrazolium sodium salt (CCK-8). Fresh medium was replaced and 100 μL of DMEM solution (containing 10 μL of CCK-8 reagent) was added to each well and the incubation would last for another 2h at 37 °C. Absorbance values were next measured at 450 nm and 650 nm.

Vero E6 cells were inoculated in 96-well plates (20 000 cells/well) and incubated with different concentrations of dihydromyricetin derivatives for 1 h, and virus (Omicron virus BA.5, MOI = 0.01) was added to infect the cells for 2 h^38^. Then, the medium containing virus and compound was removed and freshly prepared medium containing compound was added to incubate the cells. After 24 h post-infection, cell supernatants were collected and the viral RNA in the supernatants was analysed by fluorescence quantitative qRT-PCR^56^. The blank control was used DMSO, and the experiment and three parallel wells each time.

#### Therapeutic model

Vero E6 cells were inoculated in 96-well plates (10 000 cells/well) and placed in the incubator overnight for wall attachment. Dilute the compounds by solubilisation in DMSO to the corresponding 8 concentration gradients. Prepare one plate for normal cytotoxicity test (CC)and another for infected cytotoxicity test (EC). Prepare a 96-well deep-well plate by adding 297 μL of infection medium to each well, then add 3 μL of the above diluted compounds to each well, mix well and set aside. The cell supernatant of the 96-well cell plate was removed, the compound configured in the above steps was added, and the control hole was added with 1%DMSO medium, 100 μL per well. Then 100 μL of culture medium was added to each well of the CC plate, and 100 μL of diluted viral solution was added to the EC plate (Omicron virus BA.5, MOI = 0.05), and the total volume of the well plate was 200 μL, and the concentration of DMSO was 0.5%. The well plates were placed in the incubator for further culture for four days. The culture plates were taken out, the supernatant was removed, and 50 μL Celltiter-Glo reagent was added to measure luminescence on the enzyme labeller^57^. The CC_50_ and EC_50_ values were calculated using GraphPad Prism software. All infection experiments were performed on the condition of biosafety level 3 (BSL-3) of Wuhan Institute of Virology, CAS.

## Results and discussion

### Synthesis and characterisation

**Scheme 1.**
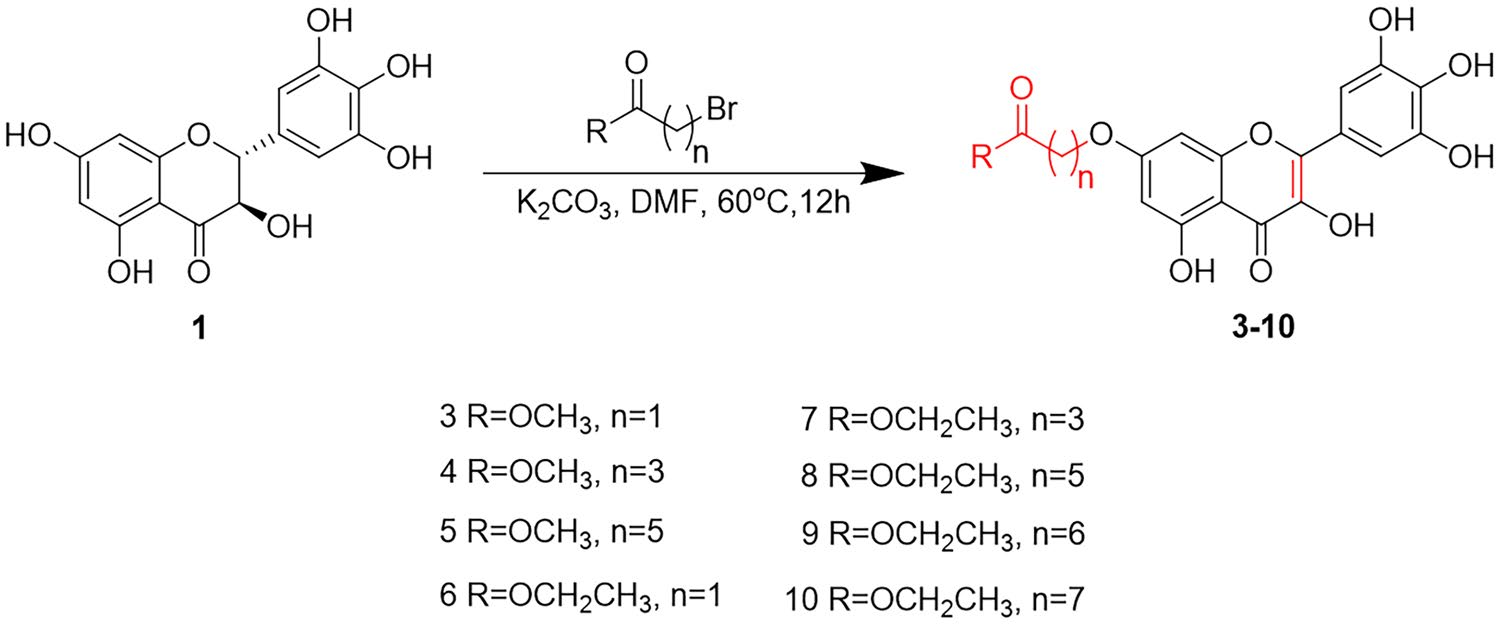
One-pot synthesis route of compounds **3**–**10**.

The chemical structures of DHM derivatives **3**–**10** and their synthetic routes are shown in Scheme 1. Compounds **3**–**10** were synthesised via Williamson ether synthesis^58^ by introducing a long chain carboxylate group at C_7_-OH, at the same time, DHM was oxidised to myricetin in the presence of alkali and oxygen^59^. The synthetic reaction process is briefly stated as follows, dihydromyricetin, K_2_CO_3_ and bromine substituent were added to a round bottom flask, which was stirred at 60 °C. After 12 h, TLC analysis showed the complete consumption of compound **1**, then the mixture was cooled to room temperature. Purification of the crude product by octadecyl silane chromatography (ODS) and afforded the desired product. and the structure of these product were characterised by ^1^H NMR, ^13^C NMR, DEPT 135° and high-resolution mass spectrum.

#### *Methyl 2-((3,5-dihydroxy-4-oxo-2–(3,4,5-trihydroxyphenyl)-4H-chromen-7yl)oxy)acetate* (3)

Compound **3** was obtained from DHM and methyl bromoacetate with 31% yield as a yellow solid; ^1^H NMR (600 MHz, DMSO-*d*_6_) *δ* 12.51 (s, 1H), 9.49 (s, 1H), 9.23 (s, 2H), 8.88 (s, 1H), 7.27 (s, 2H), 6.67 (d, *J* = 2.2 Hz, 1H), 6.38 (d, *J* = 2.2 Hz, 1H), 4.96 (s, 2H), 3.72 (s, 3H).^13^C NMR (151 MHz, DMSO-*d*_6_) *δ* 175.9, 168.7, 163.1, 160.4, 155.8, 147.6, 145.8, 136.3, 136.1, 120.7, 107.4, 104.4, 97.8, 92.5, 64.9, 52.1. HRMS (ESI) m/z: [M + H] ^+^ calcd for C_18_H_15_O_10_^+^ 391.0665; found 391.0641.

#### Methyl4-((3,5-dihydroxy-4-oxo-2–(3,4,5-trihydroxyphenyl)-4H-chromen-7-yl)oxy)butano -ate (4)

Compound **4** was obtained from DHM and methyl 4-bromobutyrate with 29% yield as a yellow solid; ^1^H NMR (600 MHz, DMSO-*d_6_*) *δ* 12.49 (s, 1H), 9.45 (s, 1H), 9.21 (s, 2H), 8.87 (s, 1H), 7.27 (s, 2H), 6.64 (d, *J* = 2.1 Hz, 1H), 6.32 (d, *J* = 2.1 Hz, 1H), 4.11 (t, *J* = 6.4 Hz, 2H), 3.61 (s, 3H), 2.48 (d, *J* = 7.3 Hz, 2H), 2.00 (p, *J* = 6.9 Hz, 2H).^13^C NMR (151 MHz, DMSO-*d_6_*) *δ* 175.8, 172.9, 164.0, 160.4, 156.0, 147.4, 145.7, 136.2, 136.0, 120.7, 107.3, 104.0, 97.7, 92.2, 67.4, 51.4, 29.8, 24.0. HRMS (ESI) m/z: [M + H] ^+^ calcd for C_20_H_19_O_10_^+^ 419.0978; found 419.0957.

#### *Methyl 6-((3,5-dihydroxy-4-oxo-2–(3,4,5-trihydroxyphenyl)-4H-chromen-7-yl)oxy) hexano-ate* (5)

Compound **5** was obtained from DHM and methyl 6-bromohexanoate with 30% yield as a yellow solid; ^1^H NMR (600 MHz, DMSO-*d*_6_) *δ* 12.48 (s, 1H), 9.44 (s, 1H), 9.21 (s, 2H), 8.86 (s, 1H), 7.28 (s, 2H), 6.63 (d, *J* = 2.1 Hz, 1H), 6.31 (d, *J* = 2.1 Hz, 1H), 4.08 (t, *J* = 6.5 Hz, 2H), 3.58 (s, 3H), 2.33 (t, *J* = 7.4 Hz, 2H), 1.73 (p, *J* = 6.7 Hz, 2H), 1.59 (p, *J* = 7.2 Hz, 2H), 1.42 (p, *J* = 7.2, 6.7 Hz, 2H). ^13^C NMR (151 MHz, DMSO-*d*_6_) *δ* 175.9, 173.4, 164.3, 160.4, 156.1, 147.4, 145.8, 136.2, 136.1, 120.8, 107.4, 103.9, 97.8, 92.2, 68.3, 51.3, 33.2, 28.1, 25.0, 24.2. HRMS (ESI) m/z: [M + H] ^+^ calcd for C_22_H_23_O_10_^+^ 447.1291; found 447.1265.

#### *Ethyl 2-((3,5-dihydroxy-4-oxo-2–(3,4,5-trihydroxyphenyl)-4H-chromen-7-yl)oxy)acetate* (6)

Compound **6** was obtained from DHM and ethyl bromoacetate with 32% yield as a yellow solid; ^1^H NMR (600 MHz, DMSO-*d*_6_) *δ* 12.50 (s, 1H), 9.43 (s, 1H), 9.13 (s, 3H), 7.25 (s, 2H), 6.65 (d, *J* = 2.2 Hz, 1H), 6.35 (d, *J* = 2.2 Hz, 1H), 4.92 (s, 2H), 4.17 (q, *J* = 7.1 Hz, 2H), 1.20 (t, *J* = 7.1 Hz, 3H).^13^C NMR (151 MHz, DMSO-*d*_6_) *δ* 175.9, 168.2, 163.1, 160.4, 155.8, 147.6, 145.8, 136.3, 136.1, 120.7, 107.4, 104.4, 97.8, 92.5, 65.0, 60.9, 14.1.HRMS (ESI) m/z: [M + H] ^+^ calcd for C_19_H_17_O_10_^+^ 405.0822; found 405.0794.

#### *Ethyl 4-((3,5-dihydroxy-4-oxo-2–(3,4,5-trihydroxyphenyl)-4H-chromen-7-yl)oxy)butano -ate* (7)

Compound **7** was obtained from DHM and ethyl 4-bromobutyrate with 28% yield as a yellow solid;^1^H NMR (600 MHz, DMSO-*d*_6_) *δ* 12.48 (s, 1H), 9.44 (s, 1H), 9.20 (s, 2H), 8.88 (s, 1H), 7.28 (s, 2H), 6.63 (d, *J* = 2.1 Hz, 1H), 6.32 (d, *J* = 2.1 Hz, 1H), 4.11 (t, *J* = 6.4 Hz, 2H), 4.07 (q, *J* = 7.1 Hz, 2H), 2.46 (t, *J* = 7.3 Hz, 2H), 1.99 (p, *J* = 6.9 Hz, 2H), 1.18 (t, *J* = 7.1 Hz, 3H).^13^C NMR (151 MHz, DMSO-*d*_6_) *δ* 175.8, 172.5, 164.0, 160.4, 156.0, 147.4, 145.7, 136.2, 136.0, 120.7, 107.3, 104.0, 97.7, 92.1, 67.5, 60.0, 30.0, 24.0, 14.1. HRMS (ESI) m/z: [M − H] ^−^ calcd for C_21_H_19_O_10_^-^ 431.0978; found 431.0961.

#### *Ethyl 6-((3,5-dihydroxy-4-oxo-2–(3,4,5-trihydroxyphenyl)-4H-chromen-7-yl)oxy)hexane -ate* (8)

Compound **8** was obtained from DHM and ethyl 6-bromohexanoate with 33% yield as a yellow solid; ^1^H NMR (600 MHz, DMSO-*d*_6_) *δ* 12.48 (s, 1H), 9.44 (s, 1H), 9.20 (s, 2H), 8.87 (s, 1H), 6.64 (d, *J* = 2.1 Hz, 1H), 6.31 (d, *J* = 2.1 Hz, 1H), 4.08 (t, *J* = 6.4 Hz, 2H), 4.05 (q, *J* = 7.1 Hz, 2H), 2.31 (t, *J* = 7.4 Hz, 2H), 1.74 (p, *J* = 6.7 Hz, 2H), 1.59 (p, *J* = 7.5 Hz, 2H), 1.42 (p, *J* = 7.2 Hz, 2H), 1.17 (t, *J* = 7.1 Hz, 3H). ^13^C NMR (151 MHz, DMSO-*d*_6_) *δ* 175.8, 172.9, 164.3, 160.3, 156.0, 147.3, 145.7, 136.2, 136.0, 120.7, 107.3, 103.9, 97.8, 92.1, 68.3, 59.7, 33.4, 28.1, 24.9, 24.2, 14.2. HRMS (ESI) m/z: [M − H] ^−^ calcd for C_23_H_23_O_10_^−^ 459.1291; found 459.1262.

#### *Ethyl 7-((3,5-dihydroxy-4-oxo-2–(3,4,5-trihydroxyphenyl)-4H-chromen-7-yl)oxy)heptano-ate* (9)

Compound **9** was obtained from DHM and ethyl 7-bromoheptanoate with 26% yield as a yellow solid; ^1^H NMR (600 MHz, DMSO-*d*_6_) *δ* 12.48 (s, 1H), 9.44 (s, 1H), 9.21 (s, 2H), 8.86 (s, 1H), 7.27 (s, 2H), 6.63 (d, *J* = 2.2 Hz, 1H), 6.32 (d, *J* = 2.2 Hz, 1H), 4.08 (t, *J* = 6.5 Hz, 2H), 4.04 (q, *J* = 7.1 Hz, 2H), 2.29 (t, *J* = 7.3 Hz, 2H), 1.72 (p, *J* = 6.8 Hz, 2H), 1.54 (p, *J* = 7.2 Hz, 2H), 1.40 (q, *J* = 7.6 Hz, 2H), 1.33 (q, *J* = 7.1 Hz, 2H)), 1.17 (t, *J* = 7.1 Hz, 3H).^13^C NMR (151 MHz, DMSO-*d*_6_) *δ* 175.8, 172.9, 164.3, 160.4, 156.0, 147.3, 145.7, 136.2, 136.0, 120.7, 107.3, 103.9, 97.8, 92.1, 68.3, 59.7, 33.4, 28.2, 28.1, 25.1, 24.4, 14.2. HRMS (ESI) m/z: [M + H] ^+^ calcd for C_24_H_27_O_10_^+^ 475.1604; found 475.1580.

#### *Ethyl 8-((3,5-dihydroxy-4-oxo-2–(3,4,5-trihydroxyphenyl)-4H-chromen-7-yl)oxy)octan-oate* (10)

Compound **10** was obtained from DHM and ethyl 8-bromooctanoate with 25% yield as a yellow solid; ^1^H NMR (600 MHz, DMSO-*d*_6_) *δ* 12.48 (s, 1H), 9.43 (s, 1H), 9.19 (s, 2H), 8.90 (s, 1H), 7.28 (s, 2H), 6.64 (d, *J* = 2.1 Hz, 1H), 6.32 (d, *J* = 2.1 Hz, 1H), 4.08 (t, *J* = 6.5 Hz, 2H), 4.04 (q, *J* = 7.1 Hz, 2H), 2.27 (t, *J* = 7.4 Hz, 2H), 1.72 (p, *J* = 6.8 Hz, 2H), 1.53(p, *J* = 7.2 Hz, 2H), 1.40 (p, *J* = 6.9 Hz, 2H), 1.36–1.31 (m, 2H), 1.31–1.26 (m, 2H), 1.16 (t, *J* = 7.1 Hz, 3H).^13^C NMR (151 MHz, DMSO-*d*_6_) *δ* 175.8, 172.9, 164.3, 160.3, 156.0, 147.3, 145.7, 136.2, 136.0, 120.7, 107.3, 103.9, 97.8, 92.1, 68.4, 59.6, 33.5, 28.4, 28.3, 28.3, 25.2, 24.4, 14.1. HRMS (ESI) m/z: [M − H] ^−^ calcd for C_25_H_27_O_10_^−^ 487.1604; found 487.1583.

### SARS-CoV-2 3CL^pro^ inhibition assays

**Figure 2.**
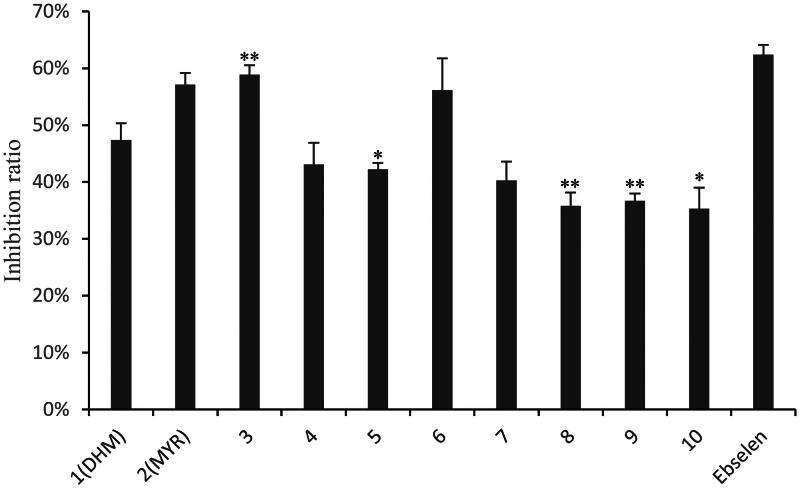
Inhibitory effect of compounds **1**–**10** against 3CL^pro^ at 1.0 μM. Note: *n* = 3, mean ± SD. Compared with the DHM: *p < .05 and **p < .01.

The inhibitory activity of the DHM derivatives against SARS-CoV-2 3CL^pro^ was measured by a fluorescence resonance energy transfer (FRET) assay. The IC_50_ value of myricetin against 3CL^pro^ is less than 1.0 μM, then the concentration was screened at 1.0 μM against 3CL^pro^ and assayed the 3CL^pro^ inhibitory activity according to the requirements on the kit (Beyotime, China). The results showed that the rate of compounds **3** and **6** inhibiting SARS-CoV-2 3CL^pro^ was more than 50% at 1.0 μM (Figure 2), which suggested that it was conducive to improve the activity by introducing a moderate length carbon chain. The dose-dependent curves of these eight compounds against SARS-CoV-2 3CL^pro^ were established, which showed that the IC_50_ of compound **3** against 3CL^pro^ was 0.72 ± 0.04 μM, it was better than that of DHM and MYR, and was comparable to the positive control drug Ebselen^54^, the IC_50_ of compounds **4**–**9** was in the range of 1.0–2.0 μM, and the IC_50_ of compound **10** was more than 2.0 μM (Table 1).

**Table 1.** Molecular docking score and IC_50_ of compounds **1**–**10**^a^ with 3CL^pro^.

3CL^pro^	Compounds	Molecular docking score						Assays
		Binding energy (kcal/mol)	H-bond number	Amino acid	Ligand	Interaction	Distance	IC_50_^b^ ± SD (μM)
PDB ID:6LU7	1 (DHM)	−6.90	6	LEU141(A)	O18	H-donor	2.81	1.35 ± 0.14
				ASN142(A)	O19	H-donor	2.81	
				CYS145(A)	O22	H-donor	3.70	
				HIS164(A)	O22	H-donor	2.88	
				PHE140(A)	O23	H-donor	3.26	
				GLY143(A)	O17	H-acceptor	3.12	
	2 (MYR)	−7.54	7	LEU141(A)	O19	H-donor	2.59	0.84 ± 0.07
				ASN142(A)	O20	H-donor	2.66	
				GLU166(A)	O21	H-donor	2.79	
				GLU166(A)	O22	H-donor	2.80	
				GLN189(A)	O23	H-donor	3.16	
				GLY143(A)	O17	H-acceptor	3.41	
				SER144(A)	O19	H-acceptor	3.24	
	3	−8.46	5	LEU141(A)	O19	H-donor	2.50	0.72 ± 0.04
				ASN142(A)	O20	H-donor	2.92	
				GLU166(A)	O21	H-donor	3.32	
				GLU166(A)	O22	H-donor	2.75	
				GLY143(A)	O17	H-acceptor	3.37	
	4	−7.76	6	SER 144(A)	O19	H-donor	3.48	1.26 ± 0.09
				THR26 (A)	O21	H-donor	3.30	
				THR26 (A)	O22	H-donor	2.77	
				THR190(A)	C29	H-donor	3.32	
				SER144(A)	O17	H-acceptor	2.86	
				GLN189(A)	O30	H-acceptor	3.47	
	5	−8.87	5	ASN142(A)	C12	H-donor	2.84	1.69 ± 0.16
				ASN142(A)	O20	H-donor	2.76	
				MET165(A)	C28	H-donor	3.77	
				GLY143(A)	O17	H-acceptor	3.17	
				CYS145(A)	O17	H-acceptor	3.51	
	6	−7.69	6	THR190(A)	O2	H-donor	2.72	0.95 ± 0.12
				THR190(A)	O3	H-donor	2.86	
				CYS145(A)	O6	H-donor	3.81	
				HIS164(A)	O6	H-donor	2.99	
				MET165(A)	C24	H-donor	3.43	
				CYS145(A)	C26	H-donor	4.03	
	7	−7.71	4	LEU141(A)	O4	H-donor	2.77	1.53 ± 0.14
				SER144(A)	O4	H-donor	2.76	
				THR26(A)	O6	H-donor	2.79	
				ASN142(A)	C29	H-donor	3.15	
	8	−7.75	3	GLU166(A)	O2	H-donor	2.69	1.73 ± 0.11
				GLU166(A)	O3	H-donor	2.65	
				ASN142(A)	O4	H-donor	3.04	
	9	−7.93	6	ASN142(A)	O2	H-donor	2.79	1.68 ± 0.19
				GLU166(A)	O3	H-donor	2.83	
				PHE140(A)	O4	H-donor	2.86	
				THR25(A)	O33	H-acceptor	3.50	
				HIS41(A)	O6	H-pi	3.23	
				CYS145(A)	6-ring	pi-H	3.94	
	10	−8.08	3	GLU166(A)	O2	H-donor	2.87	2.36 ± 0.13
				GLU166(A)	O3	H-donor	2.65	
				GLU166(A)	O8	H-donor	3.00	
	Ebselen							0.65 ± 0.01

^a^ Compound **1**: Dihydromyricetin (DHM); compound **2**: Myricetin (MYR); 3CL^pro^: SARS-CoV-2 3CL^pro^.

^b^ IC_50_ values are averages of three independent experiments.

**Figure 3.**
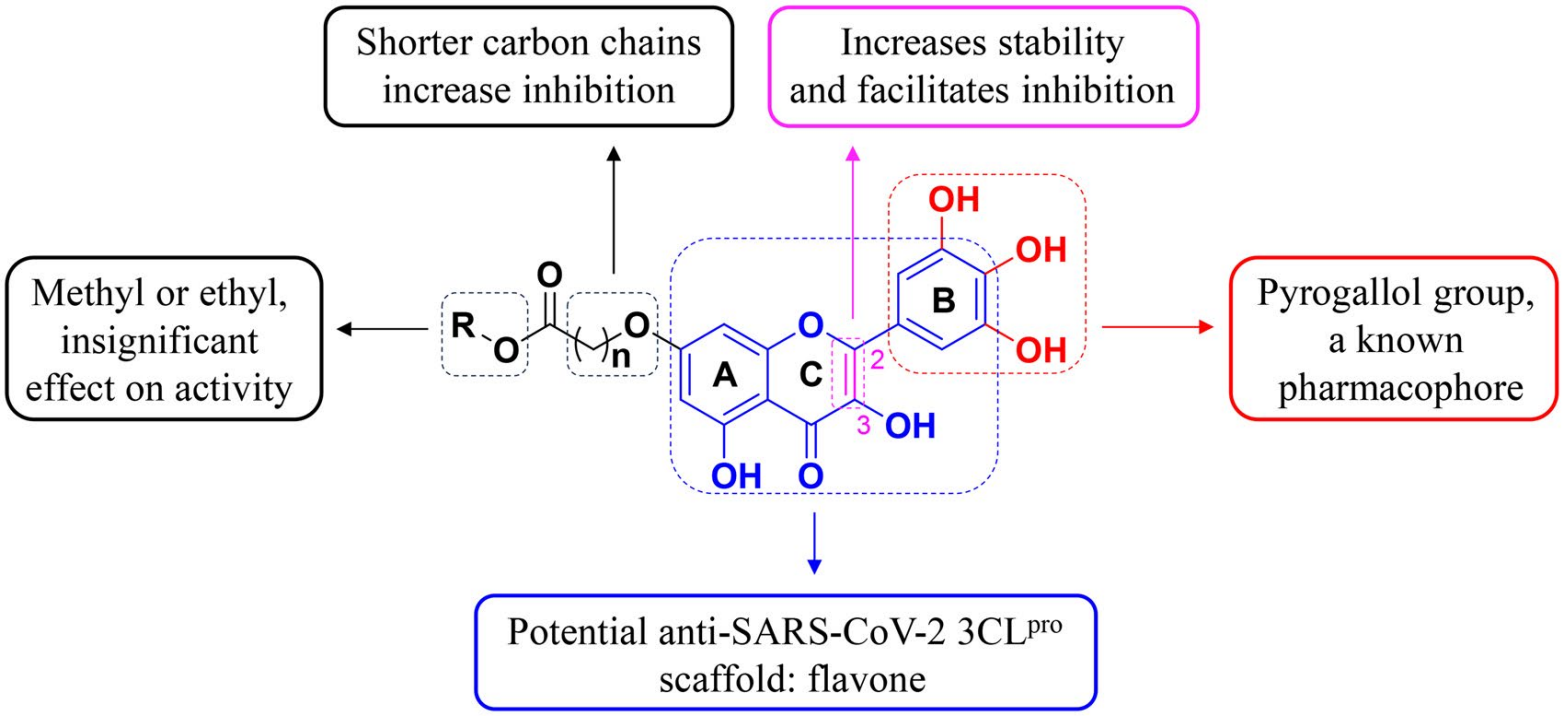
SAR of dihydromyricetin derivatives inhibition SARS-CoV-2 3CL^pro^.

The SAR of C_7_-OH were verified based on IC_50_ and the inhibition values. Compounds **3**–**5** were different length chain methyl ester and compounds **6**–**10** were different length chain methyl ester respectively. Whether derivatives were methyl ester or ethyl ester at the end, it did not obviously effect on the inhibitory activity of the 3CL^pro^, it seemed that the activity was related to the whole alky length of the introduced moiety, and the shorter the carbon chain became, the better to inhibit SARS-CoV-2 3CL^pro^ (Figure 3).

### Molecular docking

**Figure 4.**
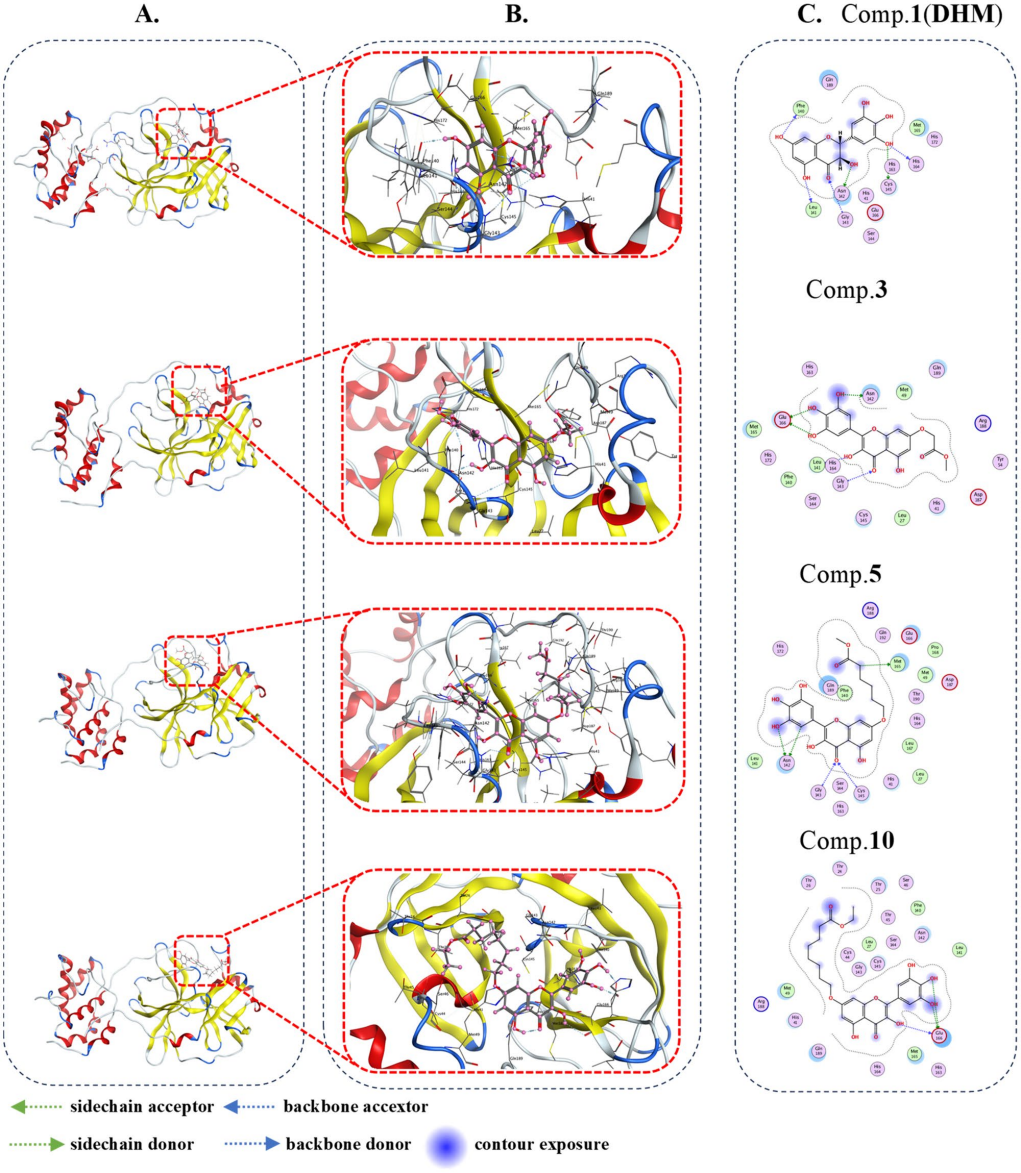
Molecular docking of compounds **1**(**DHM**), **3**, **5,** and **10** with 3CL^pro^. (A) Schematic diagram, (B) 3D enlargement, and (C) 2D schematic of the hydrogen-bond interaction between compounds **1, 3, 5, 10,** and 3CL^pro^, respectively.

Simulated molecular docking showed that eight derivatives entered the crystal structure of 3CL^pro^ (shown in Table 1), the binding energies of compounds **3**–**10** to 3CL^pro^ were −8.46, −7.76, −8.87, −7.69, −7.71, −7.75, −7.93 and −8.08 kcal/mol respectively. Briefly, DHM (Comp. **1**) formed hydrogen bonds with Leu141, Asn142, Cys145, His164, Phe140 and Gly143 (Figure 4, Comp. **1**). Comp. **3** formed hydrogen bonds with Leu141, Asn142, Glu166 and Gly143 (Figure 4, Comp. **3**). Comp. **5** formed hydrogen bonds with Asn142, Met165, Gly143 and Cys145 (Figure 4, Comp. **5**). Comp. **10** formed hydrogen bonds with Glu166 (Figure 4, Comp. **10**). These results indicated that the bound of compounds **3** to 3CL^pro^ was tighter relatively, and compound **3** could form hydrogen bonds with LEU141, ASN142, GLU166 and GLY143, and the O19, and it is closer to LEU141 with lower binding energy, compared to DHM and myricetin. These results suggested that the eight compounds could insert into the 3CL^pro^ pocket and formed hydrogen bonds with the amino acids, thereby interfering with SARS-CoV-2 replication.

To further visualise the effect of DHM derivatives with 3CL^pro^, molecular dockings were made detailly about compounds **3**, **5**, **10** and 3CL^pro^ respectively, and the effect of the compounds forming hydrogen bonds with nearby amino acids were shown in Figure 4. Based on simulated molecular docking binding energies and 3CL^pro^ inhibition assays, compounds **3**, **5**, and **10** were selected for further evaluation of the base and cellular anti-SARS-CoV-2 assays. The reasons for selecting the three compounds were as follows. (1) The simulated binding energy respectively of these three compounds was less than −8.00 kcal/mol, and the simulated binding energy of compound **5** to 3CL^pro^ was the lowest. (2) IC_50_ value of compound **3** against 3CL^pro^ was comparable to that of Ebselen. (3) Proper lipophilicity is necessary for a compound to cross the cell membrane, and compound **10** has the longest carbon chain among all derivatives.

### Anti-SARS-CoV-2 activity in Vero E6 cells

#### Preventive model

**Figure 5.**
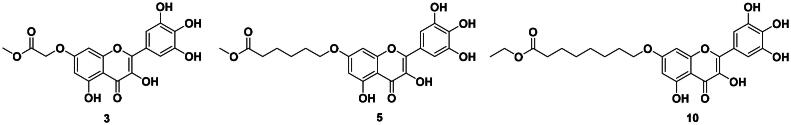
The chemical structure of compounds **3, 5,** and **10**.

Compounds **3**, **5** and **10** (Figure 5) were evaluated for activity against SARS-CoV-2 in the preventive model. The CC_50_ of the compounds against normal Vero E6 cells was first determined, indicating that the cytotoxicity of these compounds was very low. Subsequently, different concentrations of compounds were incubated with E6 cells for 1 h. Then, the virus (Omicron virus BA.5, MOI = 0.01) was added to infect the cells for 2 h. After removing the supernatant, the compound-containing medium was added to continue the incubation for 24 h. The antiviral effect was evaluated by real-time fluorescence quantitative RT-PCR (qRT-PCR). DHM, MYR and Remdesivir^60^ were used as controls (Table 2).

**Table 2.** Antiviral activity and cytotoxicity of dihydromyricetin derivatives against SARS-CoV-2 Omicron virus in the preventive model and therapeutic model.

Compound	Preventive model		Therapeutic model	
	EC_50_^a^ (μM)	CC_50_^b^ (μM)	EC_50_ (μM)	CC_50_ (μM)
**1 (DHM)**	21.25 ± 0.62	>100	ND^c^	>100
**2 (MYR)**	12.25 ± 0.23	>100	ND	>100
**3**	15.84 ± 0.92	>100	ND	>100
**5**	37.55 ± 2.05	>100	ND	>100
**10**	46.15 ± 4.67	>100	11.52 ± 3.68	53.81 ± 0.98
**Remdesivir**	0.64 ± 0.12	>100	0.91 ± 0.39	49.70 ± 0.13

^a^Concentration for 50% reduction in viral infectivity.

^b^Concentration that reduces the number of viable cells by 50%.

^c^Not detectable, inactive at 10 μM. All data are shown as mean ± SD.

#### Therapeutic model

Compounds **3**, **5** and **10** were further evaluated for activity against SARS-CoV-2 in the therapeutic model. Prepare one plate for normal cytotoxicity test (CC) and another for infected cytotoxicity test (EC). Different concentrations of compounds were added to Vero E6 cells along with the virus (Omicron virus BA.5, MOI= 0.05) and incubated for four days, the supernatant was removed, and 50 μL Celltiter-Glo reagent was added to measure luminescence on the enzyme labeller. DHM, MYR and Remdesivir were used as controls (Table 2).

Dose-dependent inhibition of Omicron virus replication by these three derivatives in the preventive model, which showed that compounds **2**(MYR) and **3** against Omicron virus was better than DHM. EC_50_ of compound **3** against SARS-CoV-2 virus (Omicron virus BA.5) in Vero E6 cells was 15.84 ± 0.92 μM in the preventive model. In the therapeutic model, 10 μM DHM and myricetin did not have significant inhibitory activity, but compound **10** showed moderate inhibitory activity against Omicron virus with the EC_50_ = 11.52 ± 3.68 μM. The experimental data of compounds **1**(DHM)**, 2(**MYR) **3**, **5**, **10** and Remdesivir against Omicron virus are shown detailly in Table 2. The results showed that the oxidation of DHM into myricetin not only improved the stability, but also nearly doubled the inhibitory activity in the preventive model. The SAR of DHM derivatives on 3CL^pro^ are consistent with prevention modelling results. The longer the carbon chain decreased the preventive activity but increased the therapeutic activity when it was modified with etherification reaction at C_7_-OH. It is possible that the higher lipophilicity is more conducive for the compounds to penetrate the cell membranes and thus exert antiviral activity at the cellular level in the therapeutic model^38^. In the molecular docking rendering, when compounds **1**(**DHM**), **3** or **5** are docked with 3CL^pro^, the compound binds to the docking pocket in an inward stereoscopic configuration, however, compound **10** bounds in an outward stereoscopic configuration (Figure 4), such that compound 10 exhibits inhibitory activity in the therapeutic model. Binding of DHM derivatives to 3CL^pro^ results in the loss of cleavage of the coronavirus polyprotein, and the virus is unable to undergo subsequent translation and replication, and thus unable to continue to produce daughter viruses. Therefore, DHM and its derivatives showed preventive and therapeutic effects against Omicron virus.

## Conclusion

In order to enhance the lipophilicity and biological activity of DHM, an oxidising and substituting one-pot strategy has been developed to synthesise DHM derivatives and eight novel DHM derivatives were obtained. All of these derivatives were effective in inhibiting SARS-CoV-2 3CL^pro^, and the structure-activity relationship indicated that the shorter carbon chain introduced at C_7_-OH favoured the inhibition of SARS-CoV-2 3CL^pro^. Molecular docking showed that the binding energy of these derivatives to 3CL^pro^ was much lower than that of DHM and myricetin. Anti-SARS-CoV-2 assays were performed at cellular level and constructed two models including a preventive model and a therapeutic model. The results showed that the longer the carbon chain decreased the preventive activity but increased the therapeutic activity when it was modified by etherification reaction at C_7_-OH. In the Vero E6-cell, compound **3** has a good activity of anti-SARS-CoV-2 virus (Omicron virus BA.5) in the prevention model, with an EC_50_ of 15.84 ± 0.92 μM, and so do compound **10** in the therapeutic model, with an EC_50_ of 11.52 ± 3.68 μM.

In conclusion, DHM and its derivatives are still inhibitory to Omicron virus, and the experimental data support DHM as a prophylactic agent against Omicron virus. It is also worth mentioning that DHM is abundantly found in Meicha, which is a very rich natural resource; more than 12 sites in the DHM molecule are considered to be chemically active, and therefore a large number of derivatives can be designed and synthesised to optimise the insufficient pharmacokinetic and pharmacodynamic properties of DHM. Most importantly, it is highly recommended that extensive investigations and studies carefully are to be conducted to evaluate the efficacy and safety of DHM against Omicron viruses and the prevention of all types of Omicron virus infections.

## Supplementary Material

Supplementary Material.docx

## Data Availability

Complete data for all compounds that support the findings of this study are available from the corresponding author upon reasonable request.
