# Supplementary material for: Design, synthesis, and biological activity evaluation of dihydromyricetin derivatives against SARS-CoV-2-Omicron virus
Source: J Enzyme Inhib Med Chem. 2024 Aug 29;39(1):2390909. doi: 10.1080/14756366.2024.2390909 (PMC11363738; doi:10.1080/14756366.2024.2390909)
Supplement: Supplementary Material.docx [file IENZ_A_2390909_SM9998.docx]

Supporting Information

**Design, Synthesis, and Biological Activity Evaluation of Dihydromyricetin Derivatives against SARS-CoV-2-Omicron Virus**

Cong Wu^a^, Qi Jiang^a^, Hui Zhong^a^, Xudong Zhou^a,b^, Leping Liu^b^, Tong Pan^a^, Chao Liu^c^,

Wei Wang^a,b*^, Wenbing Sheng^a,b*^

^a^*School of Pharmacy, Hunan University of Chinese Medicine, Changsha, Hunan 410208, People’s Republic of China;*

^b^*TCM and Ethnomedicine Innovation and Development International Laboratory, Hunan University of Chinese Medicine, Changsha, Hunan 410208, People’s Republic of China;*

*^c^**Zhangjiajie Meicha Technology Research Center Hunan Qiankun Biotechnology Co., Ltd, Zhangjiajie 427000, Peoples Republic of China.*

* Corresponding author

**List of Contents**

^1^H-NMR spectrum (600 MHz) of compound **3**S4

^13^C-NMR spectrum (151 MHz) of compound **3**S4

DEPT 135˚ spectrum (151 MHz) of compound **3**S5

HRMS spectrum of compound **3**S5

^1^H-NMR spectrum (600 MHz) of compound **4**S6

^13^C-NMR spectrum (151 MHz) of compound **4**S6

DEPT 135˚ spectrum (151 MHz) of compound **4**S7

HRMS spectrum of compound **4**S7

^1^H-NMR spectrum (600 MHz) of compound **5**S8

^13^C-NMR spectrum (151 MHz) of compound **5**S8

DEPT 135˚ spectrum (151 MHz) of compound **5**S9

HRMS spectrum of compound **5**S9

^1^H-NMR spectrum (600 MHz) of compound **6**S10

^13^C-NMR spectrum (151 MHz) of compound **6**S10

DEPT 135˚ spectrum (151 MHz) of compound **6**S11

HRMS spectrum of compound **6**S11

^1^H-NMR spectrum (600 MHz) of compound **7**S12

^13^C-NMR spectrum (151 MHz) of compound **7**S12

DEPT 135˚ spectrum (151 MHz) of compound **7**S13

HRMS spectrum of compound **7**S13

^1^H-NMR spectrum (600 MHz) of compound **8**S14

^13^C-NMR spectrum (151 MHz) of compound **8**S14

DEPT 135˚ spectrum (151 MHz) of compound **8**S15

HRMS spectrum of compound **8**S15

^1^H-NMR spectrum (600 MHz) of compound **9**S16

^13^C-NMR spectrum (151 MHz) of compound **9**S16

DEPT 135˚ spectrum (151 MHz) of compound **9**S17

HRMS spectrum of compound **9**S17

^1^H-NMR spectrum (600 MHz) of compound **10**S18

^13^C-NMR spectrum (151 MHz) of compound **10**S18

DEPT 135˚ spectrum (151 MHz) of compound **10**S19

HRMS spectrum of compound **10**S19

^1^H NMR spectra of compound **3**

^13^C NMR spectra of compound **3**

DETP 135°spectra of compound **3**


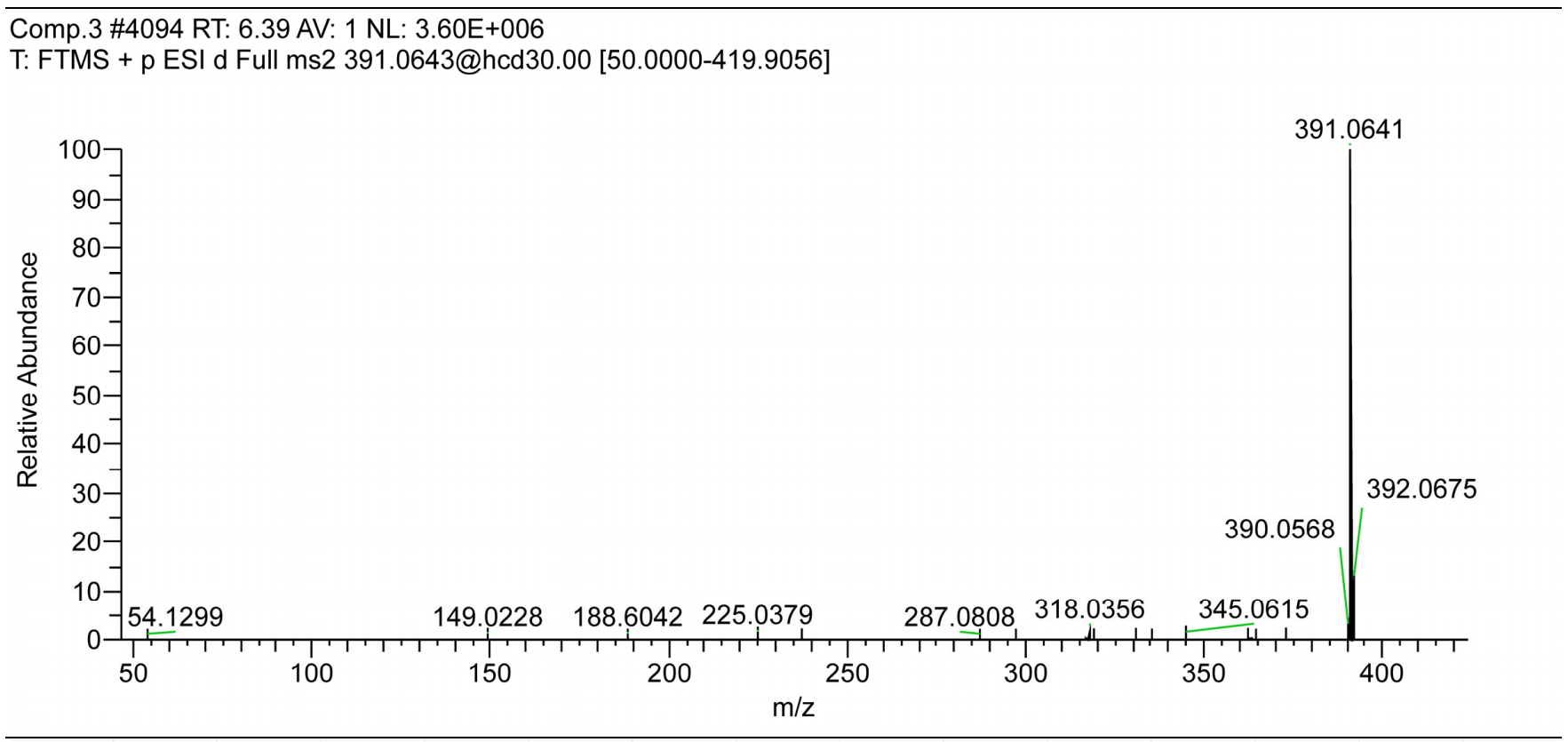


HRMS spectra of compound **3**

^1^H NMR spectra of compound **4**

^13^C NMR spectra of compound **4**

DETP 135°spectra of compound **4**


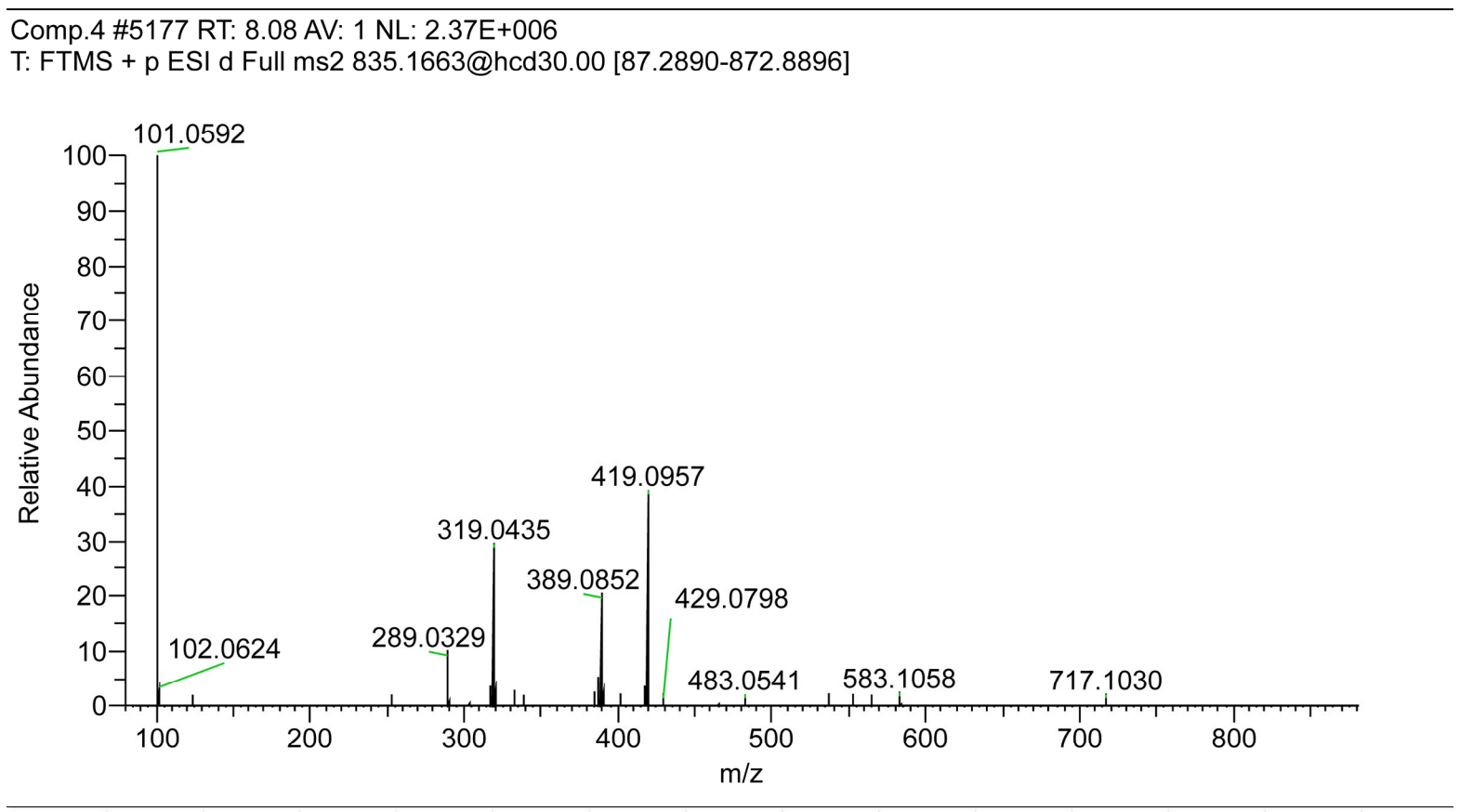


HRMS spectra of compound **4**

^1^H NMR spectra of compound **5**

^13^C NMR spectra of compound **5**

DETP 135°spectra of compound **5**


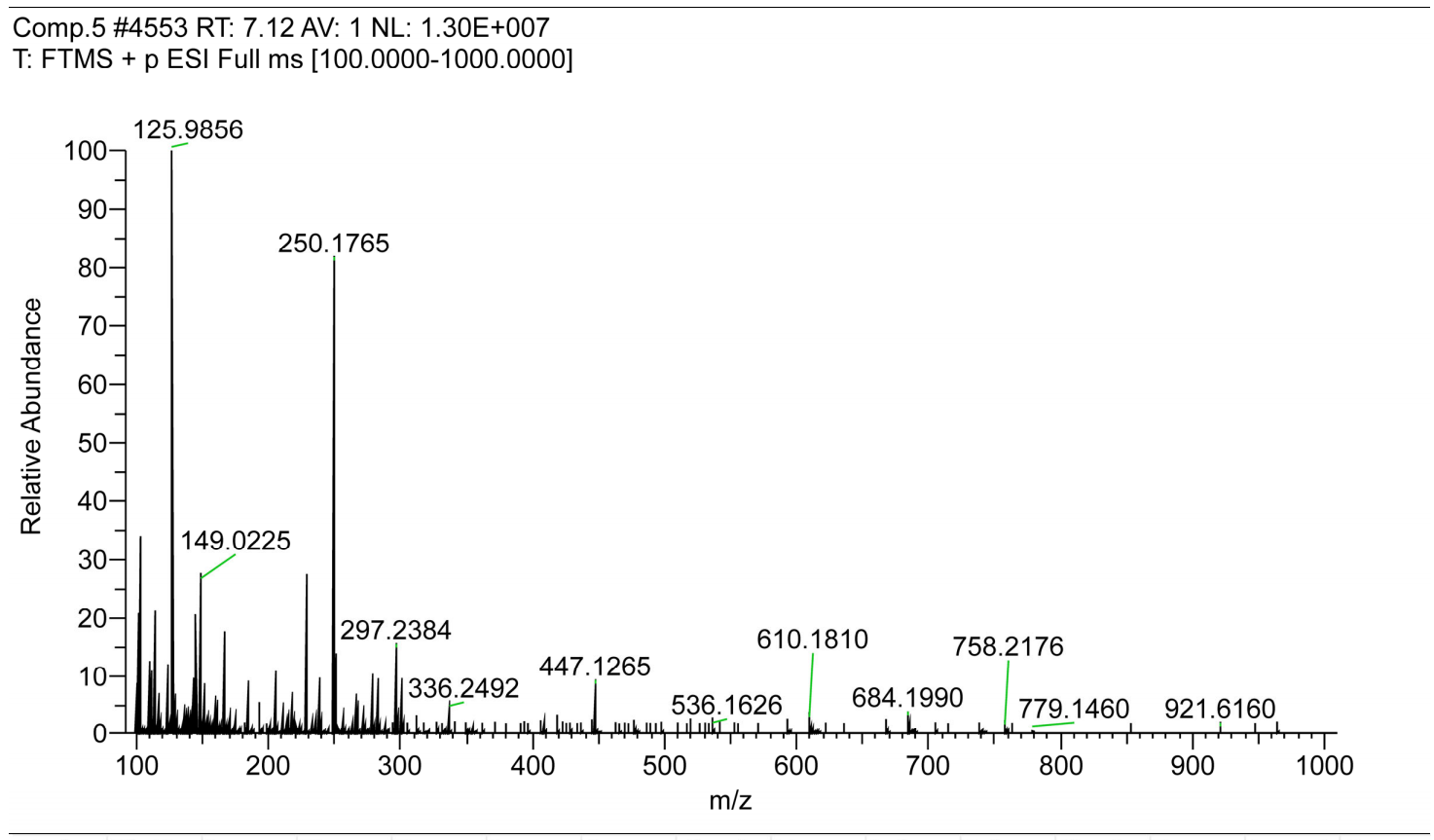


HRMS spectra of compound **5**

^1^H NMR spectra of compound **6**

^13^C NMR spectra of compound **6**

DETP 135°spectra of compound **6**


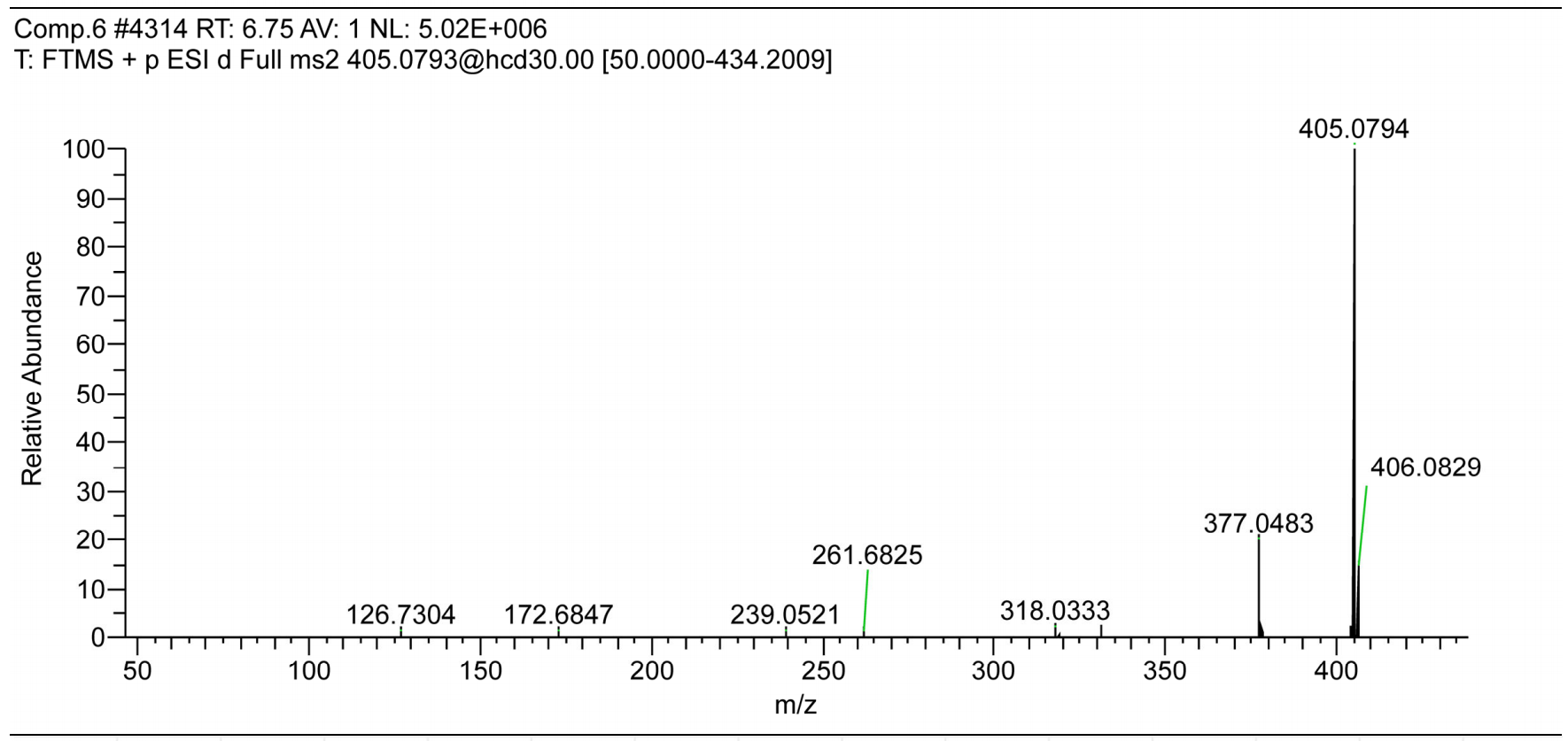


HRMS spectra of compound **6**

^1^H NMR spectra of compound **7**

^13^C NMR spectra of compound **7**

DETP 135°spectra of compound **7**


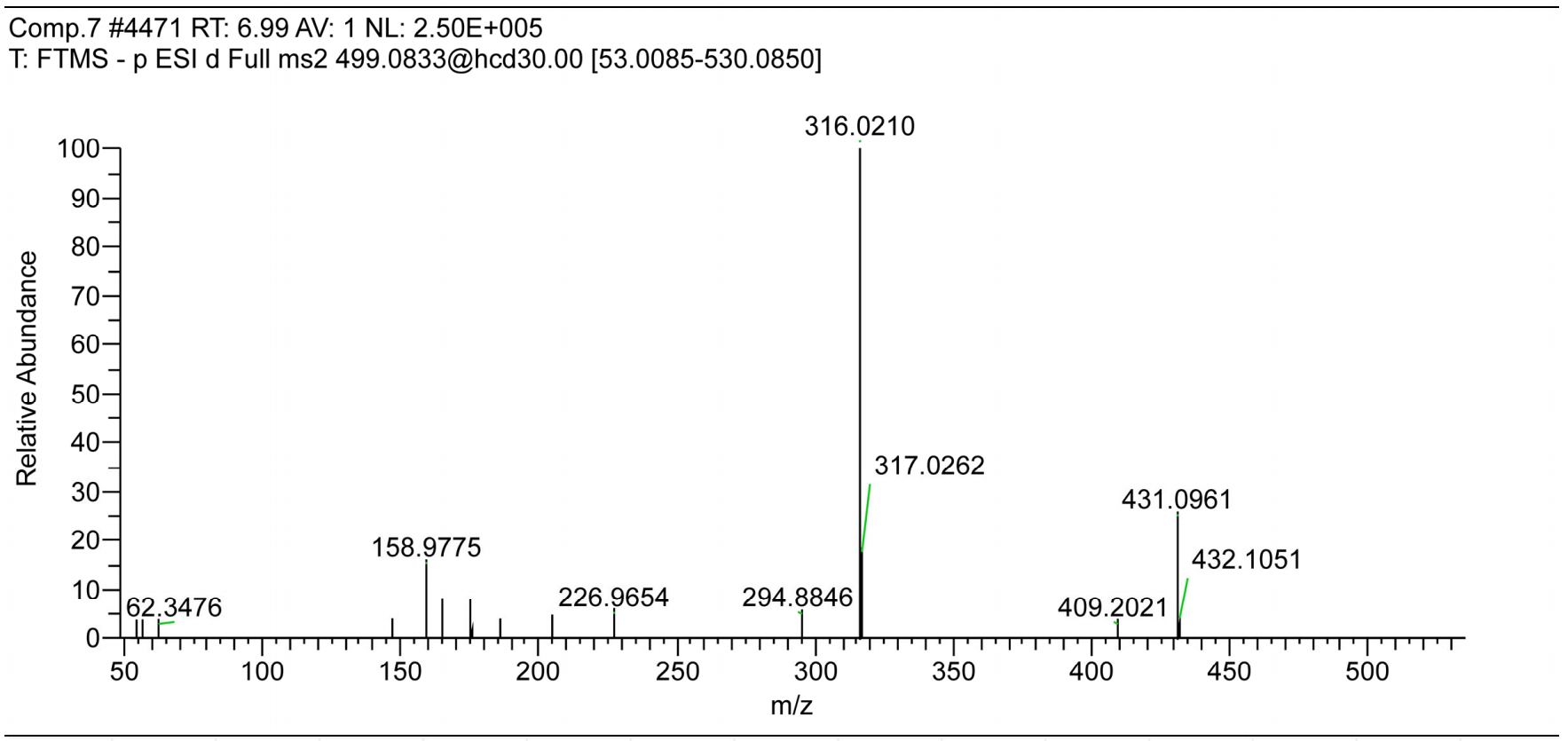


HRMS spectra of compound **7**

^1^H NMR spectra of compound **8**

^13^C NMR spectra of compound **8**

DETP 135°spectra of compound **8**


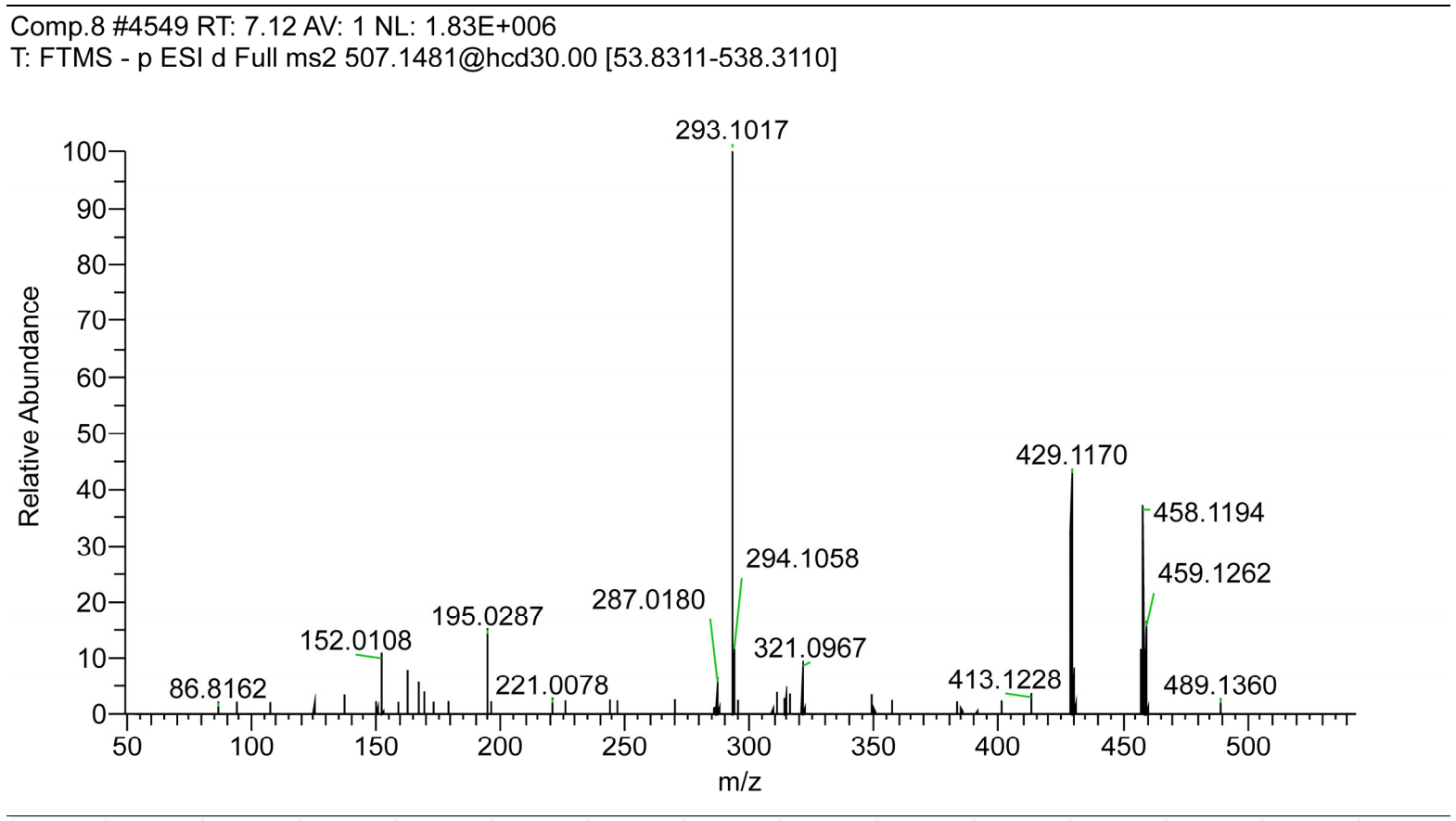


HRMS spectra of compound **8**

^1^H NMR spectra of compound **9**

^13^C NMR spectra of compound **9**

DETP 135°spectra of compound **9**


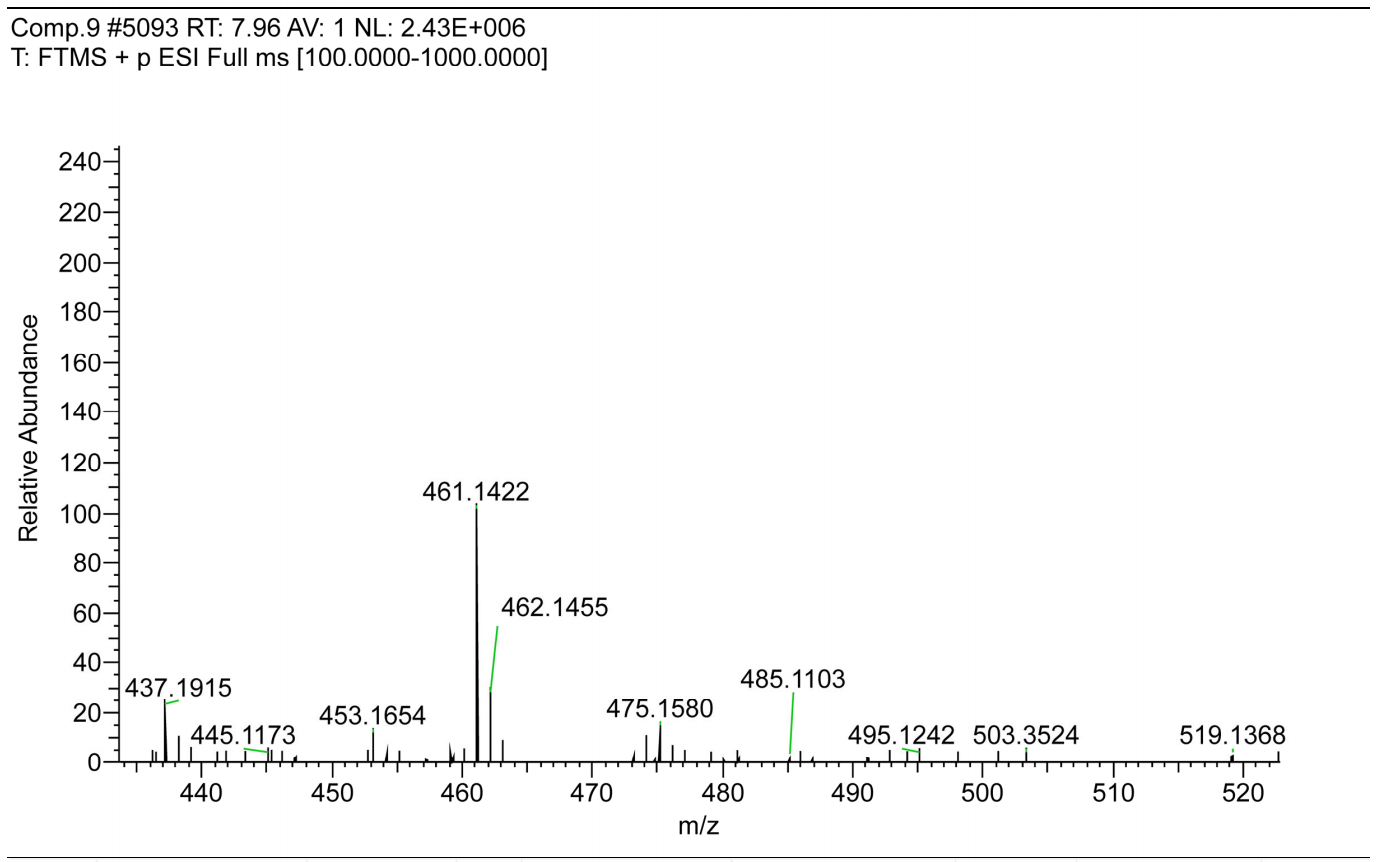


HRMS spectra of compound **9**

^1^H NMR spectra of compound **10**

^13^C NMR spectra of compound **10**

DETP 135°spectra of compound **10**


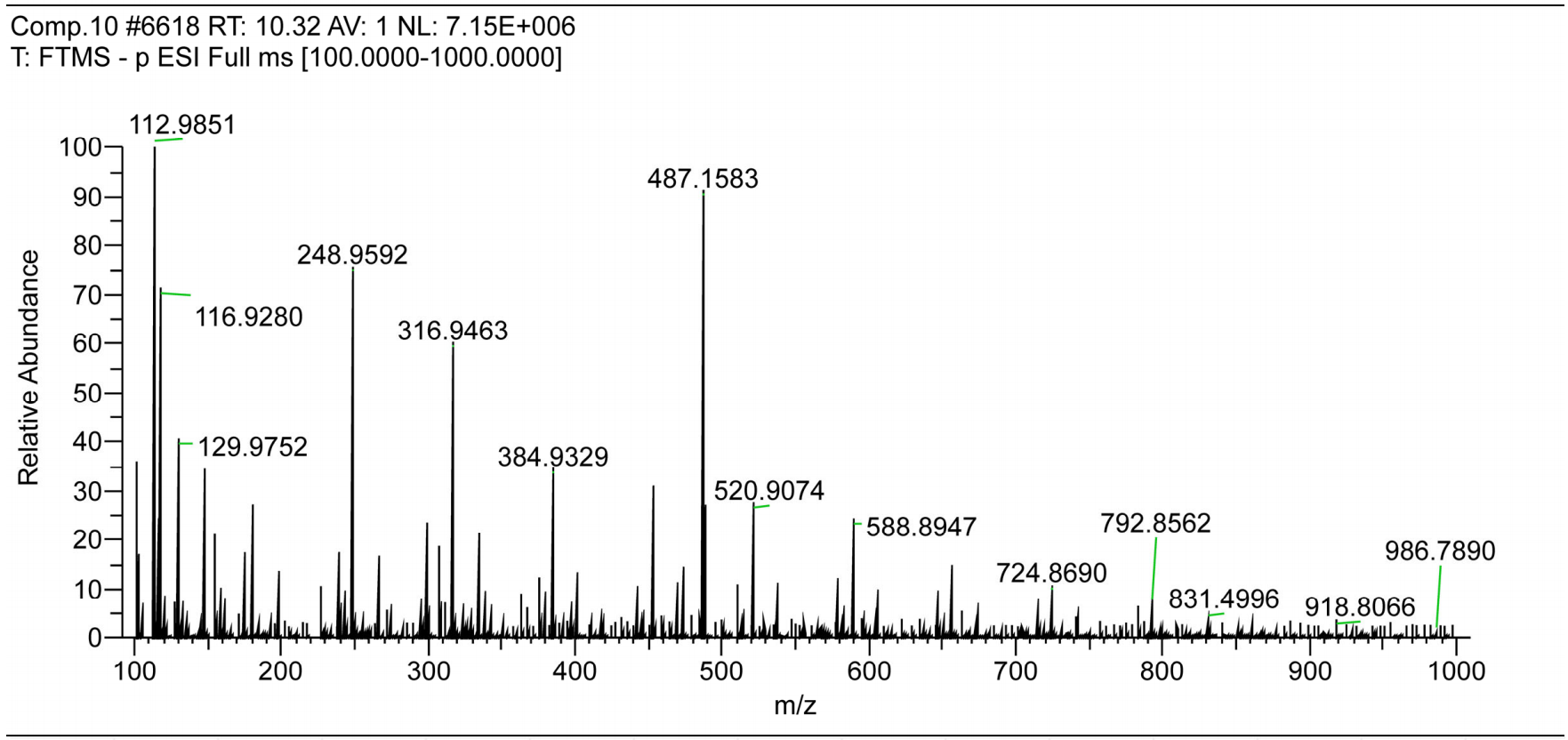


HRMS spectra of compound **10**
